# The Pyrin Inflammasome in Health and Disease

**DOI:** 10.3389/fimmu.2019.01745

**Published:** 2019-08-07

**Authors:** Oskar Schnappauf, Jae Jin Chae, Daniel L. Kastner, Ivona Aksentijevich

**Affiliations:** Metabolic, Cardiovascular and Inflammatory Disease Genomics Branch, National Human Genome Research Institute, National Institutes of Health, Bethesda, MD, United States

**Keywords:** autoinflammatory diseases, familial Mediterranean fever, pyrin inflammasome, RhoA GTPases, serine-threonine kinase, *Yersinia* toxins

## Abstract

The pyrin inflammasome has evolved as an innate immune sensor to detect bacterial toxin-induced Rho guanosine triphosphatase (Rho GTPase)-inactivation, a process that is similar to the “guard” mechanism in plants. Rho GTPases act as molecular switches to regulate a variety of signal transduction pathways including cytoskeletal organization. Pathogens can modulate Rho GTPase activity to suppress host immune responses such as phagocytosis. Pyrin is encoded by *MEFV*, the gene that is mutated in patients with familial Mediterranean fever (FMF). FMF is the prototypic autoinflammatory disease characterized by recurring short episodes of systemic inflammation and is a common disorder in many populations in the Mediterranean basin. Pyrin specifically senses modifications in the activity of the small GTPase RhoA, which binds to many effector proteins including the serine/threonine-protein kinases PKN1 and PKN2 and actin-binding proteins. RhoA activation leads to PKN-mediated phosphorylation-dependent pyrin inhibition. Conversely, pathogen virulence factors downregulate RhoA activity in a variety of ways, and these changes are detected by the pyrin inflammasome irrespective of the type of modifications. *MEFV* pathogenic variants favor the active state of pyrin and elicit proinflammatory cytokine release and pyroptosis. They can be inherited either as a dominant or recessive trait depending on the variant's location and effect on the protein function. Mutations in the C-terminal B30.2 domain are usually considered recessive, although heterozygotes may manifest a biochemical or even a clinical phenotype. These variants are hypomorphic in regard to their effect on intramolecular interactions, but ultimately accentuate pyrin activity. Heterozygous mutations in other domains of pyrin affect residues critical for inhibition or protein oligomerization, and lead to constitutively active inflammasome. In healthy carriers of FMF mutations who have the subclinical inflammatory phenotype, the increased activity of pyrin might have been protective against endemic infections over human history. This finding is supported by the observation of high carrier frequencies of FMF-mutations in multiple populations. The pyrin inflammasome also plays a role in mediating inflammation in other autoinflammatory diseases linked to dysregulation in the actin polymerization pathway. Therefore, the assembly of the pyrin inflammasome is initiated in response to fluctuations in cytoplasmic homeostasis and perturbations in cytoskeletal dynamics.

## Introduction

**Figure 1:**
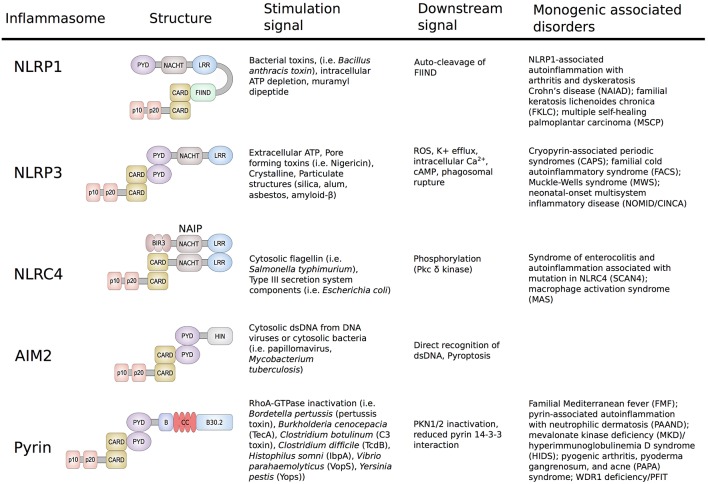
Schematic representation of the structure and function of NLRP1, NLRP3, NLRC4/IPAF, AIM2, and Pyrin Inflammasomes. Shown are the minimal NLRP1, NLRP3, NLRC4/IPAF, AIM2, and pyrin inflammasomes, their corresponding stimulation signals, described resulting downstream effects and associated disorders. Formation of the inflammasome initiates autocatalytic activation of caspase-1, resulting in cleavage of the pro-enzyme into the active p20 and p10 subunits. The active enzyme is assembled in the form of heterodimers and cause release of mature IL-1β/IL-18 and gasdermin D cleavage.

The innate immune system forms molecular platforms to recognize components of pathogenic bacteria and to differentiate these danger signals from host motifs. The cells that form this first line of defense against pathogenic bacteria, namely macrophages, monocytes, dendritic cells, and neutrophils, express a variety of pattern recognition receptors (PRRs) that detect pathogen-associated molecular patterns (PAMPs). The membrane-bound family of Toll-like receptors (TLRs) is the most extensively studied group of PRRs and recognizes PAMPs in the extracellular milieu and in different types of intracellular endosomes (1). Signaling through these receptors leads to the expression of proinflammatory cytokine-inducing transcription factors, such as NF-κB. Additionally, TLR signaling triggers the activation of interferon regulatory factors that mediate the type I interferon-dependent antiviral response. A second set of pathogen recognition sensors is present in the cytosol and includes the family of nucleotide-binding domain leucine-rich repeat (NLR) proteins (NLRP1, NLRP3, NLRP7, and NLRC4), the protein absent in melanoma 2 (AIM2), and pyrin. These sensors are essential for detection of pathogens and endogenous danger-associated molecular patterns (DAMPs) inside the cell and their activation triggers the formation of multiprotein complexes, called inflammasomes (Figure 1) (2, 3).

NLRP1 was the first cytosolic sensor identified to form a caspase-1 activating inflammasome in response to the virulence factor lethal toxin produced by *Bacillus anthracis* (4). The antigen component of this toxin forms a membrane-inserted pore through which the anthrax lethal factor is delivered to the host cytosol. Upon cell entry, the anthrax lethal factor induces assembly and activation of the NLRP1 inflammasome. As NLRP1 plays a major role in host innate immune response, unsurprisingly, malfunctions in this inflammasome were shown to cause disease. Several common and low-penetrance polymorphisms in the *NLRP1* gene were associated with a number of autoimmune disorders, including vitiligo, systemic lupus erythematosus, inflammatory bowel disease, and celiac disease (5–7). The important role of NLRP1 was further highlighted in two publications from 2016 that for the first-time linked novel high-penetrance variants in *NLRP1* to human Mendelian monogenic disease (Figure 1) (8, 9).

The identification of the NLRP3 inflammasome was a major breakthrough in the field of innate immunity and autoinflammation (10). In 2004, Agostini et al. (10) showed that increased activity of the NLRP3 inflammasome is the molecular basis of the symptoms in patients with cryopyrin-associated periodic syndromes (CAPS). This study demonstrated that dominantly inherited gain-of-function (GOF) mutations in *NLRP3* cause activation of caspase-1, and an excessive release of IL-1β, which subsequently led to the recognition of IL-1 receptor antagonists and other IL-1 inhibitors as successful therapies for these disorders. NLRP3 is the best studied inflammasome and many distinct signals have been found to cause its activation including adenosine triphosphate (ATP), pore-forming bacterial toxins, crystalline and particulate structures, as well as cathepsin B released from lysosomes. The variety of activating stimuli indicates that the assembly of the NLRP3 inflammasome is likely stimulated through a common downstream signal. To date, several distinct unifying signals such as potassium efflux, mitochondrial reactive oxygen species (ROS), increased intracellular Ca^2+^, and decreased cellular cyclic AMP (cAMP) have been proposed (8–10). However, the detailed mechanism of NLRP3 inflammasome activation has yet to be determined.

In contrast to NLRP3, the NLRC4/IPAF inflammasome responds to a limited set of stimuli and is triggered by virulence factors produced by Gram-negative pathogens known as type III (T3SS) and IV (T4SS) secretion systems (11, 12). The mechanism of NLRC4 activation is unique amongst inflammasomes in that it requires binding of another NLR-family member, the neuronal apoptosis inhibitor proteins (NAIPs). NAIPs act as sensors for PAMPs and upon direct binding to the ligands, NAIPs associate with NLRC4 to form the NAIP/NLRC4 inflammasome (Figure 1). This in turn induces recruitment and activation of caspase-1 and subsequently release of mature IL-1β and IL-18. Phosphorylation of NLRC4 by protein kinase Cδ is a critical event for inflammasome formation and was shown to induce a conformational change of NLRC4 (13). In 2014, two independent groups reported that novel and/or *de novo* GOF mutations in *NLRC4* cause autoinflammatory syndromes with distinct features of infantile enteropathy and macrophage activation syndrome (MAS) (14, 15).

The AIM2 protein is composed of a N-terminal pyrin domain (PYD) and a C-terminal HIN-200 domain and in contrast to NLRPs lacks the NLR/NACHT domain. Direct binding of cytosolic double stranded DNA (dsDNA) to the HIN-200 domain of AIM2 induces its release of the autoinhibitory conformation and allows inflammasome assembly and activation (16). To date, no GOF mutations in *AIM2* that cause human inherited autoinflammatory disease have been described. One possible explanation is that in contrast to NLR-containing inflammasomes that can form independently from ligands, AIM2-oligomerization is dependent of direct binding to DNA molecules.

The NLRP7 inflammasome has not been well-characterized, particularly in comparison to the other inflammasomes. One of the few described activation signals are microbial acylated lipopeptides that were shown to induce an ASC-dependent caspase-1 activation of NLRP7 (17). Interestingly, in addition to its proinflammatory role, NLRP7 also possesses properties that inhibit inflammation. Suggested mechanisms for its anti-inflammatory role include interaction with proteins that repress NF-κB signaling as well as direct sequestration of pro-caspase-1 and pro-IL-1β (18). Biallelic rare loss-of-function (LOF) mutations in *NLRP7* have been associated with recurrent hydatiform mole (19, 20).

Even though the presented inflammasomes differ in components and pattern recognition, they are all unified in their capability to mediate activation of caspase-1, which promotes the maturation of the proinflammatory cytokines IL-1β and IL-18 and the induction of inflammatory cell death (pyroptosis). Pyroptosis is morphologically different from apoptosis in that it involves cell swelling and lysis. Pyroptosis involves caspase-1 mediated cleavage of gasdermin D (GSDMD), subsequent translocation of the N-terminal pore-forming domain to the cellular membrane and release of pro-inflammatory cytokines (21, 22). Pyroptosis can also be triggered by direct binding of LPS to caspase-11 in mouse cells and caspase-4 and 5 in human cells, which results in caspase oligomerization and cleavage of GSDMD. Cleavage of gasdermin E by caspase-3 was also shown to induce pyroptosis (23, 24). Thus, pyroptosis plays a major role in amplifying the protective immune responses during an infection (25).

Dysregulation or erroneous activation of the described inflammasomes can lead to autoinflammatory diseases, a group of genetically diverse but symptomatically similar disorders. Variability in clinical manifestations can be explained by cell-specific functions of these proteins. For example, NLRC4 is highly expressed in epithelial cells while NLRP1 is more abundant in keratinocytes, thus GOF mutations lead to severe inflammation in gastrointestinal and skin, respectively. In contrast to conventional autoimmune disorders, autoinflammatory diseases are not primarily mediated by the cells of adaptive immunity such as antigen-specific T-cells or antibodies producing B-cells. They are therefore considered the Mendelian disorders of the innate immunity (26).

## Identification and Structure of Pyrin

**Figure 2:**
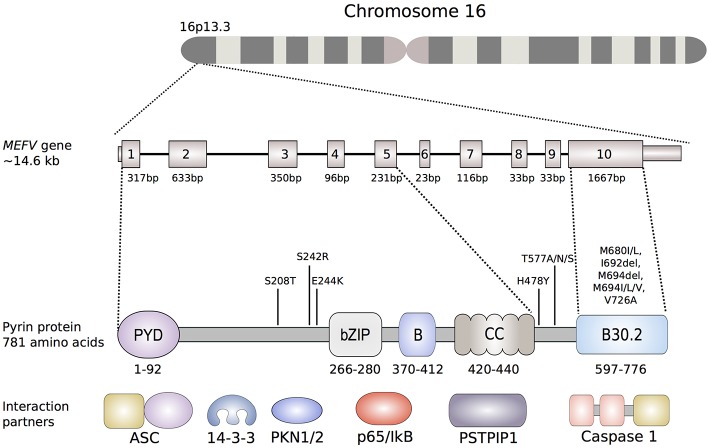
Schematic representation of the *MEFV* gene and the encoded pyrin protein. Of the more than 300 nucleotide variants described in the *MEFV* gene, only shown are the ones that are clearly associated to disease phenotypes. The most common FMF-associated mutations reside in exon 10, that encodes the B30.2 domain (B30.2). Mutations in the B30.2 domain tend to be transmitted in an autosomal-recessive fashion, while the mutations in exons 2, 3 and 5 more often exhibit an autosomal-dominant pattern of inheritance. Known pyrin interaction partners are depicted next to their corresponding or putative interaction domain of the pyrin protein: ASC (apoptosis-associated speck-like protein containing a CARD), 14-3-3 (14-3-3 protein), PKN1/2 (serine-threonine kinases PKN1 and PKN2), p65 (transcription factor p65), IκB (NF-κB inhibitor), PSTPIP1 (proline serine threonine phosphatase-interacting protein).

The pyrin protein (also known as marenostrin; TRIM20), named after the Greek word for fever, is a 781-amino acid, ~95 kDa protein that is encoded by *MEFV* on chromosome 16 (Figure 2). Pyrin expression is mainly confined to the cells of the innate immune system, namely granulocytes, eosinophils, monocytes, and dendritic cells. Homology analyses identified five different domains within the pyrin protein (Figure 2). The eponymous PYD domain (1–92) at the N-terminal end of the protein is found in more than 20 human proteins that are mainly involved in inflammatory processes. Via its PYD domain, the protein binds to the inflammasome adaptor protein, apoptosis-associated speck-like protein with a caspase recruitment domain (ASC), which subsequently causes caspase-1 mediated production of IL-1β (27).

Due to the presence of a bZIP transcription factor domain (266–280) and of two overlapping nuclear localization signals, early structural analyses suggested a nuclear function for pyrin (28). This hypothesis was further supported by a study that demonstrated that a variant protein, lacking a domain encoded by exon 2, indeed translocated to the nucleus and that the N-terminal fragment of pyrin interacts with the p65 subunit of NF-κB (29, 30). However, later studies investigating localization and function of pyrin found that full-length pyrin is mainly located in the cytosol and that the N-terminal half of pyrin colocalizes with both microtubules and the actin cytoskeleton (31). Furthermore, the localization of pyrin was also shown to be dependent on the expressing cell type and further research is needed to decipher the possible cell-type specific functions of pyrin (32).

The B-box (370–412) and the α-helical, coiled-coil (420–440) domain may play a role in the oligomerization of pyrin (33). These two domains were also shown to interact with the proline serine threonine phosphatase-interacting protein (PSTPIP1/CD2BP1), a protein that is important for the organization of the cytoskeleton (34). Missense mutations in *PSTPIP1/CD2BP1* cause a dominantly inherited autoinflammatory syndrome called pyogenic arthritis, pyoderma gangrenosum, and acne (PAPA) (35).

The C-terminal B30.2 domain of pyrin is of particular importance since most of the FMF-associated mutations are clustered in this domain and it is therefore essential for the molecular mechanisms leading to FMF. *In vitro* overexpression studies showed that the B30.2 domain of pyrin directly interacts with caspase-1. However, studies investigating the effect of FMF-associated mutations on the binding affinity of B30.2 to caspase-1 led to conflicting results (36, 37).

## Familial Mediterranean Fever

FMF, the prototypic autoinflammatory disease, is characterized by recurrent episodes of fever with serosal inflammation manifesting with severe abdominal or chest pain, arthralgia, monoarticular arthritis and limited erythematous skin rash. The onset of symptoms is typically in childhood and the episodes of fever with abdominal/chest pain usually resolve within 48–72 h. Laboratory findings of FMF resemble an attack of acute inflammation with elevated erythrocyte sedimentation rate and C-reactive protein, leukocytosis, thrombocytosis, as well as fibrinogen and immunoglobulins in the blood (38). Inflammation in FMF patients is well-controlled by treatment with colchicine or IL-1 inhibitors. The most severe complication of FMF is a secondary serum amyloid A (SAA) protein amyloidosis that can affect various tissues, commonly kidneys. These patients usually have severe and chronic inflammation lasting for many years and are often resistant or non-compliant to treatment with colchicine. The mortality rate was very high in the era before colchicine and IL-1 inhibitors. The incidence of SAA amyloidosis is far lower nowadays with improved health care for FMF patients in most affected populations.

## Mode of Inheritance of Familial Mediterranean Fever

In 1958, Heller et al. (39) described FMF as a genetic disease that exhibits autosomal dominant inheritance with incomplete penetrance, due to the high prevalence of disorder in the non-Ashkenazi Jewish population. Later studies with larger cohorts postulated that FMF is an autosomal recessively inherited disorder and pseudodominance was suspected as the reason for divergent results in earlier studies (40, 41). Based on these segregation analyses FMF has long been considered a recessive illness and the *MEFV* positional cloning studies were therefore based on an autosomal-recessive model of inheritance (28). *MEFV*, as the causative gene for FMF, was identified by two independent groups in 1997 (28, 42).

The advent of genetic testing and the increase in diagnosed patients led to the recognition that approximately 30% of all cases clinically diagnosed with FMF carry only one demonstrable mutation despite extensive search for a second disease-causing variant (43–45). This observation suggested that a single pathogenic mutation in *MEFV* in the presence of other genetic or environmentally permissive factors might be sufficient to trigger excessive activation of the pyrin inflammasome. In addition, it was shown that asymptomatic carriers for monoallelic FMF mutations, for instance unaffected parents of FMF patients, exhibit a biochemical phenotype such as elevated inflammatory biomarkers (46, 47). The described findings, together with the fact that a recessive model of inheritance would favor disease-associated variants that are null mutations, prompted a re-evaluation of the LOF recessive model of FMF inheritance.

FMF-associated missense mutations reside in exon 10, which encodes the B30.2/SPRY domain (Figure 2). Within this domain, an FMF mutation hot-spot is identified between amino acid residues 680 and 726, with Met680Ile, Met694Val, and Val726Ala as the most frequent disease-causing variants. The carrier frequency is as high as 10% in multiple populations in the Middle East and Mediterranean basin, raising the possibility of balancing selection.

## The Proinflammatory Role of the Pyrin Inflammasome

Early studies with mice expressing a truncated form of pyrin showed an increase in caspase-1 activation and therefore suggested an anti-inflammatory function of pyrin (48). However, later studies postulated the existence of a pyrin inflammasome and a potential proinflammatory role for pyrin, but the mechanisms that lead to its activation remained elusive (49). The first study showing that pyrin regulates IL-1 processing and release in human myeloid cells was published in 2007 (50). Subsequently in 2009, Gavrilin et al. (51, 52) postulated based on siRNA knockdown experiments that pyrin forms an inflammasome in human monocytes and transfected THP1 cells upon infection with *Francisella tularensis* and *Burkoholderia cenocepacia*. Another important series of experiments that helped to distinguish between an anti- or proinflammatory function of pyrin were performed in 2011 in animal model studies (53). Chae et al. (48) initially generated pyrin knock-out mice that lack both copies of a murine ortholog of pyrin and these mice developed normally and had no signs of inflammation, which strongly argues against a LOF model. Subsequently, pyrin knockin (KI) mice were generated with murine pyrin fused to the human B30.2 domain carrying the most common FMF mutations, Met680Ile, Met694Val, and Val726Ala (53). Because murine pyrin lacks the B30.2 domain, the generation of the fusion protein was necessary to investigate pyrin's role in the pathogenesis of FMF. Only homozygous KI mice, carrying two copies of the mutated fusion protein, developed a severe inflammatory phenotype. These findings indicate a GOF mechanism for pyrin inflammasome activation.

## Activation and Function of the Pyrin Inflammasome

The engagement of pyrin through the appropriate stimulus leads to the assembly of an inflammasome, and the subsequent activation of caspase-1 and release of IL-1β and IL-18. An important step in this process is the recruitment of ASC to pyrin. Via its N-terminal PYD domain, ASC enters a PYD-PYD homotypic interaction with pyrin, which induces its oligomerization of micrometer-sized assemblies, the ASC specks (49, 54–56). Subsequently, pro-caspase-1 is recruited to the specks via interaction of the caspase recruitment domain (CARD) of ASC with the CARD domain of the pro-caspase-1. The resulting clustering of pro-caspase-1 molecules promotes a proximity-induced autoproteolytic induction of caspase activity (3, 57). The autocleavage of pro-caspase-1 leads to the formation of active caspase-1 p10/p20 tetramer, which processes pro-IL-1β and pro-IL-18 to their mature forms. Another mechanism contributing to inflammation in FMF is GSDMD-mediated pyroptosis, which results in the release of cytoplasmic content, including mature IL-1β and IL-18 (21, 58). These cytokines then work as potent initiators and amplifiers of innate immune responses and induce a variety of defense processes including fever, hematopoiesis, lymphocyte activation, leukocyte attraction, and antibody synthesis (59). The described pyroptotic mechanisms also lead to the release of ASC specks into the extracellular space where these specks exhibit “prionoid” features to further promote the inflammatory response (55, 60).

**Figure 3:**
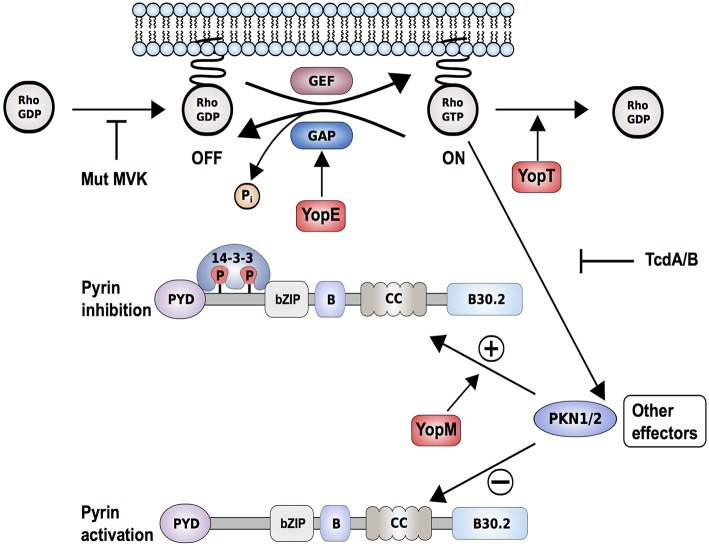
Proposed interaction model of *Yersinia pestis* effectors, Yops, with the pyrin inflammasome. *Yersinia pestis* effectors YopE (by promoting GTP hydrolysis) and YopT (by catalyzing cleavage and detachment of RhoA from the plasma membrane) inactivate RhoA. This results in reduced PKN1/2 activity, which in turn promotes the assembly of a pyrin inflammasome. Other bacterial toxins such as Clostridium difficile toxin B/TcdB (by glycosylation) or deleterious mutations in key enzymes of the mevalonate kinase pathway (Mut-MVK) also cause RhoA-inactivation. By delivering the additional effector YopM to the host cell, *Yersinia pestis* maintains virulence. YopM stimulates the PKN-1/2-mediated phosphorylation of pyrin and thereby the inhibition of pyrin inflammasome by hijacking host kinases PKN-1/2.

The type of ligands or signals that trigger pyrin activation remained unknown until 2014, when Xu et al. (61) demonstrated that pyrin can sense pathogen-induced modifications of host Rho guanosine triphosphatases (Rho GTPases). This study showed that TcdB, a virulence factor of *Clostridium difficile*, known to glycosylate and thereby downregulate the activity of a small Rho GTPase, RhoA, can activate the pyrin inflammasome (Figure 3) (62). Bone marrow-derived macrophages (BMDMs) treated with wildtype TcdB exhibit a strong pyrin-mediated inflammasome reaction and increased caspase-1 activity, resulting in pyroptosis. This effect was abolished when a glucosyltransferase-defective mutant form of TcdB was used. The described modification of RhoA is not restricted to TcdB. Other bacterial proteins, such as C3 toxin (*Clostridium botulinum*), pertussis toxin (*Bordetella pertussis*), VopS (*Vibrio parahaemolyticus*), IbpA (*Histophilus somni*), as well as the TecA toxin of *Burkholderia cenocepacia*, were also shown to add distinct modifications to the switch I region domain of RhoA (63–66). Due to the variety of post-translational modifications and the lack of direct interaction between pyrin and RhoA, it was proposed that pyrin does not directly recognize specific modifications but rather is triggered by an indirect signal downstream of RhoA. The fact that Rho GTPases control many aspects of the actin cytoskeleton dynamics led to a hypothesis that pyrin may sense changes in the cytoskeleton organization. Further support for this postulation came from studies on WDR1, a regulator of actin-cytoskeleton dynamics. Mice and humans deficient for the *WDR1* gene present with a distinct IL-1 independent, but IL-18 dependent autoinflammatory phenotype and thrombocytopenia (67). WDR1 deficiency leads to an increase in actin polymerization, and these alterations are in part detected by the pyrin inflammasome (68).

## Molecular Mechanisms of Pyrin Inflammasome Activation

Four recent publications elucidated the molecular mechanisms of pyrin regulation downstream of RhoA and demonstrated how changes in host Rho GTPase activity trigger pyrin inflammasome activation (69–72). Park et al. (70) showed that the RhoA-dependent serine/threonine-protein kinases PKN1 and PKN2 directly phosphorylate pyrin at positions Ser208 and Ser242. This results in an interaction of pyrin with the chaperone proteins 14-3-3ε and 14-3-3τ. This interaction keeps pyrin in an inactivate state and prevents the formation of an active inflammasome. The inactivation of RhoA through bacterial toxins causes a decrease in PKN1 and PKN2 activity and results in reduced levels of phosphorylated pyrin. This in turn releases pyrin from the inhibitory 14-3-3 proteins and facilitates the formation of an active pyrin inflammasome.

These findings were confirmed by Gao et al. (71) through experiments in murine BMDMs and dendritic cells. They showed that 14-3-3 protein binding to murine pyrin is dependent on phosphorylation of the corresponding residues (Ser205 and Ser241) in the murine pyrin ortholog. The stimulation with RhoA inactivating toxins causes a reduction in phosphorylated pyrin, the dissociation of 14-3-3 protein and subsequently the formation of a pyrin inflammasome complex.

Further evidence for the important role of phosphorylation in the regulation of pyrin came through the recent description of a new dominantly inherited disorder called pyrin-associated autoinflammation with neutrophilic dermatosis (PAAND) (69, 73). PAAND is caused by the amino acid substitution in exon 2 of *MEFV* at position 242 (Ser242Arg) or 244 (Glu244Lys) that are critical for PKN-mediated phosphorylation of pyrin (Figure 2). Overexpression of Ser242Arg or Glu244Lys mutated proteins in HEK293T or THP1 cells demonstrated spontaneous ASC-speck formation, higher caspase-1 activity, and increased inflammatory cell death suggesting constitutive activation of pyrin. Additional *in vitro* experiments showed that these substitutions cause reduced binding to 14-3-3 proteins and impair the self-regulatory mechanism of pyrin.

**Figure 4:**
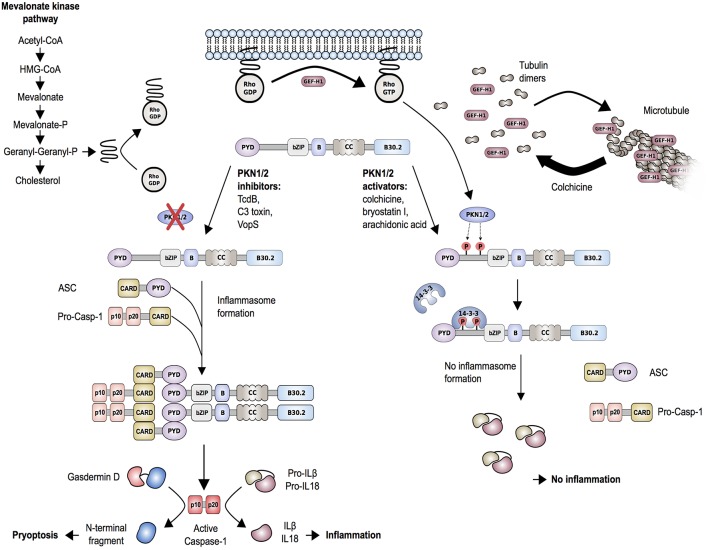
Proposed molecular mechanism of regulation of the pyrin inflammasome. Geranylgeranylation (mevalonate kinase pathway) and release of GEF-H1 (i.e., through the microtubule polymerization inhibitor colchicine) induce RhoA activity. RhoA effector kinases, PKN1 and PKN2, cause subsequent phosphorylation of pyrin and binding to inhibitory proteins 14-3-3. This mechanism is enhanced by PKN1/2 activating substances and release of GEF-H1, while low GEF-H1 or defective MVK-pathway function leads to inactivation of PKN1/2. Decreased phosphorylation of pyrin is associated with pyrin inflammasome activation and release of mature IL-1β and IL-18. The release of IL-1β and IL-18 is further facilitated by the production of the plasma membrane pore-forming N-terminal fragment of gasdermin D.

Studies of another autoinflammatory disorder caused by mevalonate kinase (MVK) deficiency provided additional support for the described mechanism of pyrin inflammasome regulation. MVK is a key enzyme of the mevalonate/cholesterol pathway and biallelic hypomorphic mutations in *MVK* cause mevalonate kinase deficiency (MKD)/hyperimmunoglobulinemia D syndrome (HIDS) (74). Besides cholesterol, the mevalonate pathway also synthesizes other intermediates, including geranylgeranyl pyrophosphate, which serves as a substrate for a specific type of post-translational lipid modification, called protein geranylgeranylation. Akula et al. (72) showed that geranylgeranylation of the small GTPase Kras is essential for the TLR-induced activation of PI3K-Akt signaling that maintains pyrin in the inhibitory state. Loss of Kras-geranylgeranylation causes an unchecked TLR-induced inflammatory response and leads to a constitutive activation of the pyrin inflammasome. RhoA is also a subject to geranylgeranylation and the translocation of RhoA from the cytosol to the cellular membrane, an essential step for its activation, is dependent on this modification (Figure 4). Park et al. (70) showed that the inhibition of the MVK pathway in BMDMs induces the release of membrane-bound RhoA and pyrin inflammasome-dependent secretion of IL-1β. The IL-1β production was blocked through the addition of geranylgeranyl pyrophosphate or through chemical activation of PKN1 and PKN2. Thus, the inflammation in patients with MKD/HIDS is mediated by the pyrin inflammasome.

The described studies demonstrated that the pyrin inflammasome does not directly interact with PAMPs, but rather indirectly senses pathogen-induced changes in RhoA activity. This indirect mechanism represents a paradigm shift for the sensing of pathogens in the mammalian immune system and resembles the “guard hypothesis” by which innate immunity is triggered by resistance proteins in plants (75). Resistance proteins recognize the downstream effects of pathogen virulence factors rather than the factors themselves and therefore fulfill a surveillance function in cellular homeostasis. The indirect pyrin inflammasome activation can therefore be considered as an example of the “guard” mechanism in humans.

## Molecular Pathogenesis of Familial Mediterranean Fever

The described studies on the molecular mechanism regulating the pyrin inflammasome also were essential to better understand the pathogenesis of FMF and how mutations in *MEFV* can lead to excessive inflammasome activation. The fact that FMF-associated mutations cluster in the B30.2 domain implies that mutations might interfere with an important regulatory role of this domain. Park et al. (70) demonstrated that binding of PKN1 to the mutant pyrin knock-in mice with common FMF-associated mutations was substantially decreased relative to binding of PKN1 to wild-type mouse pyrin, which lacks a B30.2 orthologous domain. This finding was confirmed by studies in HEK293T cells, ectopically expressing wildtype or mutated pyrin, as well as in macrophages, differentiated from PBMCs of FMF patients. In both model systems, binding of inhibitory protein 14-3-3ε to human mutant pyrin was substantially reduced relative to binding to wildtype pyrin.

These experiments indicated that binding of 14-3-3 proteins to phosphorylated pyrin acts as a molecular block to keep the pyrin inflammasome in an inactive state. Disturbances that influence 14-3-3 binding to pyrin, either through bacterial pathogens that change the phosphorylation status of pyrin or through mutations in the B30.2 domain, lead to an activation of pyrin. To date it is not clear how the B30.2 domain keeps pyrin in an inactivated state, but different hypotheses have been proposed. The B30.2 domain might function as a platform that allows binding of PKN1/2 and/or 14-3-3ε/τ and mutations in this domain interfere with the efficient docking of these proteins. A second hypothesis proposes that B30.2 domain is essential for the formation of a secondary structure that keeps pyrin in an autoinhibitory state. Phosphorylation of Ser208 and Ser242 and subsequent 14-3-3 protein binding may induce an intramolecular interaction of the B30.2 domain with the B-box/coiled-coil or other regions of pyrin. FMF-associated mutations in the B30.2 domain might result in a steric hindrance of the autoinhibition and thereby favor the active confirmation of pyrin. Interestingly, a mechanistically similar, self-inhibitory regulation was described recently for the NLRP1 inflammasome in the context of two inflammatory skin-disorders (9).

The described findings also help to better understand the mode of action of drugs used for the treatment of FMF, such as colchicine. Colchicine is an alkaloid with microtubule toxic properties and has been proven very effective as a prophylactic treatment of FMF and gout (76). Colchicine is a known RhoA activator and was shown to function through the release and thereby activation of guanine-nucleotide-exchange factor (GEF)-H1 from depolymerized microtubules (Figure 4) (77). In line with that, Park et al. (70) showed that treatment of LPS-primed BMDMs with clinically therapeutic doses of colchicine not only activated RhoA but also reversed the C3 toxin-induced inhibition of RhoA activity. Furthermore, colchicine treatment increased the interaction of 14-3-3ε to pyrin in lysates of BMDMs from mice carrying FMF-associated mutations. These findings indicate that colchicine works through the GEF-H1-dependent activation of RhoA that leads to pyrin phosphorylation and inhibition.

Interestingly, other groups proposed a microtubule-dependent and phosphorylation-independent mechanism of pyrin inflammasome activation as an alternative mechanism of action for colchicine (71, 78, 79). They confirmed the previous finding that colchicine attenuates bacterial toxin-induced caspase-1 activation, IL-1β release and pyroptosis in mouse BMDMs. However, they did not find changes in pyrin phosphorylation or 14-3-3 binding upon colchicine treatment. Gao et al. (71) showed that colchicine works downstream of pyrin phosphorylation and suggested a mechanism of action for colchicine through its inhibitory effect on ASC-speck formation. Van Gorp et al. (78) demonstrated that FMF-associated mutations allow microtubule-independent oligomerization of ASC and therefore prime pyrin for ASC-binding and inflammasome formation without microtubule-related signals.

One explanation for the discrepancies seen in colchicine downstream effects could be due to the differences in colchicine concentrations used in the respective studies. Further studies are necessary to delineate precise mechanisms of colchicine effect on the pyrin inflammasome, specifically in human cells.

Pyrin-induced pyroptosis was shown to be critical for neutrophilia and production of IL-1β in a murine model of FMF (80). Deletion of GSDMD in FMF KI mice abolished bacterial toxin-induced *ex vivo* IL-1 production from BMDMs and development of spontaneous inflammatory disease in these mice.

## Other Pyrin Inflammasome-Mediated Disorders

### Dominantly Inherited Pyrin Mediated Disorders

#### Pyrin-Associated Autoinflammation With Neutrophilic Dermatosis (PAAND)

In 2016, Masters et al. (69) identified a dominantly inherited autoinflammatory disorder, PAAND, in a three-generation Belgian family caused by a serine-to-arginine substitution at position 242 of pyrin (Ser242Arg). In contrast to FMF, patients with PAAND present with longer fever episodes and severe neutrophil-mediated dermatosis and cystic acne. Subsequently, another family with PAAND was identified and the disease-causing variant was found to be the heterozygous Glu244Lys mutation (73). Monocytes from patients with PAAND have a significantly higher spontaneous production of IL-1β and IL-18 than cells from healthy controls or FMF patients. Thus, patients with PAAND should respond to treatment with IL-1 or IL-18 inhibitors, although the latter therapy has not been evaluated yet.

Interestingly, the homozygous mutation Ser208Thr, affecting the other residue that is phosphorylated by PKN1/2, has been associated with another phenotype manifesting with failure to thrive, lymphadenopathy, transient purpuric rashes, arthralgia, oral ulcerations and mixed lymphocytic/eosinophilic infiltrates in bone marrow. Elevated levels of IL-1β and IL-18 cytokines were found in serum samples and supernatants of LPS stimulated monocytes. In addition, stimulated patients' PBMCs released increased levels of C-C-motif chemokine ligand 5 (CCL5), a potent chemotactic agent for eosinophils, which likely explains the observed eosinophilia (81). The fact that biallelic mutations that cause an amino acid change at position 208 are necessary to activate pyrin suggests that the Ser208 residue is less critical for pyrin inhibition than the Ser242 residue. Collectively, these studies have shown how identification of patients with a rare mutation can be instrumental in understanding the physiological function of a protein.

#### Pyrin-Associated Dominant Disease (PADD)

A severe autosomal-dominant periodic inflammatory disease without neutrophilic dermatosis has been reported in a three-generation family from Spain. The main findings in the five affected individuals were long fever episodes, renal amyloidosis, and colchicine resistance. All individuals were found to be heterozygous carriers for the novel pathogenic variant His478Tyr in *MEFV* (82). The His478Tyr amino acid substitution is located between the coiled-coil and the B30.2 domain of pyrin, but the exact molecular effect of this variant is still unknown (Figure 2).

Another residue in pyrin, associated with an autosomal dominant autoinflammatory syndrome, is Thr577 (83). Stoffels et al. (83) found four different heterozygous substitutions at the amino acid position 577 of pyrin in two families and two single individuals (Thr577Asn, Thr577Ala, and Thr577Ser). All patients presented with an autoinflammatory phenotype, including fever and systemic inflammation, which was similar to FMF but also showed some differences. Recently, another three-generation family of Japanese ancestry has been described carrying the Thr577Asn mutation and presenting with low-grade fevers, serositis and amyloidosis (84). PBMCs of a patient with the Thr577Asn mutation exhibit increased IL-1β secretion after LPS stimulation indicating that Thr577Asn acts through a GOF mechanism. Because of the vicinity of 577 residue to CC domains, which are known to play a role in oligomerization, it is possible that these heterozygous mutations lead to a constitutive pyrin activation, which would explain a more severe phenotype (Figure 2). The crystal structure of this region of the protein is still unresolved, therefore it is still unknown how exactly these variants cause a hyperactivation of the pyrin inflammasome.

Most FMF-associated mutations described to date are missense variants except for a couple in frame single amino-acid deletions, including Ile692del and Met694del. Interestingly, one of these variants, namely Met694del, was reported in families with dominantly inherited FMF despite intensive search for a second causal variant (85, 86).

#### Pyogenic Arthritis, Pyoderma Gangrenosum, and Acne (PAPA) Syndrome

PAPA syndrome is an autosomal dominantly inherited autoinflammatory disorder and was first described in an extended family in 1997 (87). Manifestations of PAPA syndrome encompass early-onset flares of sterile arthritis characterized by neutrophilic infiltrates. Cutaneous manifestations are variable and may include ulcerations, pyoderma gangrenosum, or cystic acne. Increased acute-phase reactants and increased production of IL-1β and TNFα in peripheral blood leukocytes are common laboratory findings in this disorder (88, 89).

Wise et al. (35) found that heterozygous mutations in CD2-binding protein 1 (*CD2BP1*), also known as proline serine threonine phosphatase-interacting protein 1 (*PSTPIP1*), are the genetic cause of PAPA syndrome. The PAPA syndrome causing mutations in *PSTPIP1* impair its association with PEST (rich in proline, glutamic acid, serine, and threonine)-type protein tyrosine phosphatase (PTP-PEST). Pyrin interacts with PSTPIP1 via its B-box/coiled-coil domain (34). The authors further demonstrated that the two most common PAPA-associated mutations (Ala230Thr and Glu250Gln) induce phosphorylation of PSTPIP1, likely due to its reduced affinity for PTP-PEST, and that the hyperphosphorylation increases the affinity of PSTPIP1 to pyrin. This may in turn induce the activation of the pyrin inflammasome and increase IL-1β production. The mechanism by which this occurs has yet to be fully understood. Pyrin and PSTPIP1 co-localize with the tubulin cytoskeleton and mutant PSTPIP1 proteins are recruited by pyrin to form ASC specs (90). Another study suggested that binding of PSTPIP1 activates pyrin by unmasking its pyrin domain, which leads to increased ASC-mediated oligomerization and inflammasome formation (33). With the identification of PKN1/2 and their role in the regulation of the pyrin inflammasome, it remains to be investigated whether binding of PKN1/2 or 14-3-3 proteins to pyrin might be affected by the differential affinity of mutant PSTPIP1. In addition, PSTPIP1 localizes to actin-rich regions in the cell and functions as an important factor for the organization of the cytoskeleton (91). This finding is particularly relevant in the view of the hypothesis that changes in cytoskeletal organization caused by invading pathogens might be one of the pyrin inflammasome triggering signals.

### Recessively Inherited Pyrin Mediated Disorders

#### Mevalonate Kinase Deficiency (MKD)/ Hyperimmunoglobulinemia D Syndrome (HIDS)

Mevalonate Kinase Deficiency (MKD)/Hyperimmuno-globulinemia D syndrome (HIDS) is a rare recessively inherited autoinflammatory disease with onset in infancy or early childhood and the first disease episode is often provoked by immunization. MKD/HIDS is characterized by recurrent fever lasting 3–7 days, cervical lymphadenopathy, abdominal pain, hepatosplenomegaly, diarrhea, arthralgia/arthritis, and erythematous maculopapular rash. Most but not all MKD/HIDS patients present with high levels of IgD, while during flares laboratory findings also include elevated urinary mevalonic acid levels and increased acute-phase reactants. The disease-associated gene *MVK* was reported in 1999 by two independent groups (92, 93). Subsequent work demonstrated that patients with nearly absent MVK enzymatic activity manifest a more severe phenotype and present with developmental disabilities and inflammation (mevalonic aciduria), while a partial deficiency causes milder, autoinflammatory MKD/HIDS (92, 94). The deficiency in MVK function seen in MKD/HIDS results in the reduction of geranylgeranyl pyrophosphate (95–97). As described earlier, reduced geranylgeranylation of Rho GTPases, RhoA, and Kras, leads to increased pyrin activity (Figure 4). As expected, the inflammation in MKD/HIDS patients is controlled with IL-1 inhibitors (98).

#### Autoinflammatory Periodic Fever, Immunodeficiency, and Thrombocytopenia (PFIT)

In 2007, a new autoinflammatory and thrombocytopenia phenotype was described in mice that is caused by a LOF mutation in the actin-depolymerizing cofactor *Wdr1* (67). Wdr1 is a WD40 repeat protein that is required for the cofilin-dependent disassembly of actin filaments and the disease-causing mutation, a T-to-A transversion in the second nucleotide of intron 9, was shown to affect mRNA-splicing. Further analyses showed that the phenotype of Wdr1 deficiency is IL-18 dependent, but IL-1β independent, and that the pyrin inflammasome is the main inflammatory mediator of this disease (68). Neutrophils and macrophages of *Wdr1*-deficient mice have elevated levels of polymerized actin compared with wild-type mice and the high levels of polymerized actin induce ASC oligomerization, caspase-1 activation, and IL-18 secretion in these cells. The treatment of these cells with latrunculin-b, a marine toxin that disrupts actin polymerization, or with colchicine, a known microtubule depolymerizing agent, caused a reduction in LPS-induced caspase-1-mediated IL-18 secretion.

**Figure 5:**
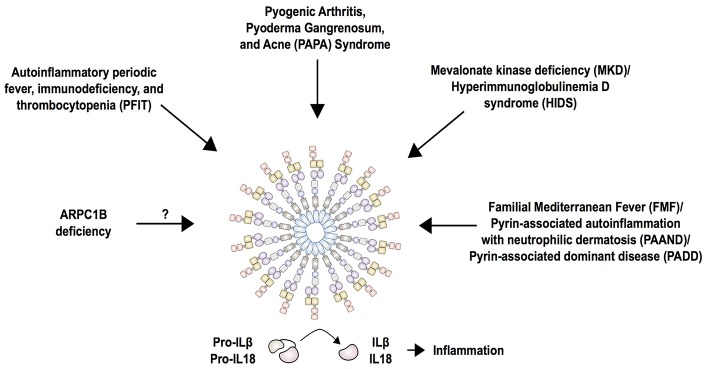
Current summary of autoinflammatory diseases mediated by the pyrin inflammasome. Pyrin interacts with ASC and caspase-1 to form the canonical inflammasome that process pro-IL-1β and pro-IL-18 into its mature forms, IL-1β and IL-18. Mutations in pyrin increase activity of the inflammasome and cause familial Mediterranean fever (FMF) and pyrin-associated autoinflammation with neutrophilic dermatosis (PAAND). While FMF mutations only enhance the activity of pyrin, PAAND-associated mutations lead to constitutive activation of pyrin, which is reflected by a higher production of IL-1 and IL-18 in PAAND than FMF. The molecular consequence of other dominantly inherited pyrin mutations (PADD) is still unknown. Mutations in MVK associated with MKD/HIDS act by downregulating RhoA activity that is important for pyrin inhibition. Mutations in PSTPIP1 (PAPA syndrome), WDR1 (PFIT), and ARPC1B affect actin polymerization, thus it is likely that these perturbations in the cytoskeleton are detected by the pyrin inflammasome.

Subsequently, homozygous missense mutations in *WDR1* were identified in two siblings who presented with autoinflammatory recurrent fevers, thrombocytopenia, and immunodeficiency. Standing et al. (99) show that LPS-stimulated patient monocyte-derived dendritic cells, CD14-lymphocytes, and Epstein-Barr virus-transformed lymphoblasts exhibit elevated levels of polymerized actin and produced higher levels of IL-18. Furthermore, HEK293T cells transfected with mutated WDR1 exhibited abnormal aggregate formation that co-localized with pyrin in fluorescent microscope analyses. These findings indicate that the association of mutated WDR1 with pyrin might cause spontaneous ASC oligomerization and pyrin inflammasome activation (Figure 5). The effect of WDR1 deficiency on neutrophil morphology, motility, and function was demonstrated in several additional affected individuals, presenting with recurrent infections, neutropenia, impaired wound healing and severe stomatitis (100). The detailed molecular mechanisms of how WDR1 deficiency leads to pyrin inflammasome activation remains to be determined.

#### ARPC1B Deficiency

Loss of function mutations in the ARPC1B subunit of actin related protein complex 2/3 (ARP2/3) have been identified in patients with early-onset immunodeficiency, low platelet count, eosinophilia, elevated IgE and IgA levels, small vessel vasculitis and predisposition to inflammatory bowel disease (101–103). The ARP2/3 complex is ubiquitous in eukaryotic cells and is essential for mitotic integrity, cell survival, and a variety of cellular functions (104). This multi-system phenotype is similar to the phenotype of patients with Wiskott-Aldrich syndrome that is caused by mutations in the WASP protein. WASP promotes actin polymerization and branching of F-actin via the ARP2/3 complex. ARPC1B expression is restricted to hematopoietic cells and consequently patients with absent or low protein expression manifest variable degrees of thrombocytopenia and immune dysregulation. The inflammatory phenotype has not yet been studied, but it is tempting to speculate that perturbations in actin polymerization might trigger an activation of the pyrin inflammasome (Figure 5). One study showed that murine *Wdr1* hypomorphic monocytes secrete significantly less IL-18 cytokine in the presence of the Arp2/3 inhibitor CK-666. The production of IL-18 was dependent on ASC, caspase-1 and pyrin. Contribution of other inflammasomes, including NLRP1, NLRP3, and AIM2, was excluded by generating double knockout/mutant mouse strains in this disease model (68).

## Evolutionary Aspects

Population studies in multiple Mediterranean populations recognized high carrier frequencies of FMF-associated mutations and suggested a selective advantage of these genotypes, probably to an endemic pathogen (105, 106). Although haplotype data showed that FMF carrier chromosomes from different ethnic groups share a common progenitor, implicating a founder effect, the unexpectedly high frequency of several distinct mutations in different populations indicates evolutionary selection. Further support for the selective advantage model comes from genetic studies of the B30.2 domain during primate evolution (107). Schaner et al. (107) showed that pyrin is not evolving at a constant rate as it would be expected for neutral evolution, but rather evolves at different rates across species. The authors hypothesized that these episodes of positive selection in different species might have been provoked by novel environmental pathogens. Moreover, wild-type pyrin of non-human primates often exhibits amino acid residues that are associated with human FMF suggesting that primates likely tolerate viral or bacterial pathogens against which mutated human pyrin confers resistance.

The recent recognition that pyrin senses changes in a variety of cellular processes might contribute to a better understanding of the proposition for selective advantage of FMF heterozygous mutations. A broad spectrum of bacterial pathogens use Rho GTPase-inactivating toxins to compromise cytoskeleton-dependent host cell defense mechanisms such as immune cell migration and phagocytosis. Other pathogenic toxins, such as the cholera toxin or pertussis toxin, alter the cytosolic concentration of cAMP to impair or to deactivate a variety of basic cellular processes and functions. These perturbations of cytoplasmic homeostasis were recently termed “homeostasis-altering molecular processes” (HAMPs) and the fact that the pyrin inflammasome senses changes of cellular homeostasis rather than directly recognizing pathogens might provide the indispensable flexibility to detect evolutionarily novel infections (108). The balancing selection of FMF-associated mutations might therefore be due to an increased “alertness” of the pyrin inflammasome for cellular changes in individuals carrying these mutations.

Along these lines, two recent publications demonstrated how pathogens develop mechanisms to evade the inflammasome-triggered immune response and showed, through the example of *Yersinia* infections, how host-pathogen co-evolution might occur (109, 110). Previous studies had shown how *Yersinia* species, including *Yersinia pestis*, the causative agent of the plague, use pathogenicity factors, so-called *Yersinia* outer proteins (Yops), to counteract multiple defense responses in the infected host cell (111). Recent work demonstrated that the pyrin inflammasome successfully recognizes *Yersinia* infections through changes in Rho GTPase activity (Figure 3). Both YopE and YopT downregulate RhoA activity and thus prevent activation of PKN1/2, which result in pyrin inflammasome activation (112). YopE and YopT-dependent pyrin activation is efficiently neutralized or hijacked by YopM, an additional Yop that is delivered into the host cell. The leucine-rich repeat (LRR)-containing protein YopM was shown to inhibit caspase-1 activation but the exact mechanism remained elusive (113). Recent findings suggested that YopM works as a scaffold protein to interact with different host proteins, including PKN1 and PKN2. By recruiting and activating these kinases, YopM facilitates the phosphorylation and inhibition of pyrin. Chung et al. (109) further speculated that the high carrier frequencies for FMF-associated variants in Mediterranean and Middle Eastern populations might have emerged because the heterozygous carriers are more protected against pathogenic *Yersinia* species.

## Perspectives and Open Questions

Following the identification of pyrin and its physiologic role many steps have been made toward a better understanding of the complexity of the pyrin inflammasome. Unequivocally, the discovery of the mechanisms of how bacterial toxins and other pathogens can indirectly trigger the pyrin inflammasome and how these signals are transmitted to downstream effectors were important achievements. However, other questions remain unanswered and new ones have arisen with these recent findings.

An important question is how the B30.2 domain regulates pyrin function and whether it acts through an autoinhibitory or proinflammatory mechanism. Murine studies implicated that the B30.2 domain has a role in autoinhibition and that disease-causing mutations confer poor affinity to regulatory 14-3-3 proteins. However, this is less clear in humans. Current consensus is that FMF-associated mutations lower a threshold for activation of the pyrin inflammasome but whether increased pyrin activity results from a loss of autoinhibition or from a facilitated activation remains unclear (114).

The major impediment in understanding the function of human pyrin and the effect of FMF mutations is in that X-ray crystallography has failed to solve the complete pyrin structure, due to the protein insolubility. Admittedly, crystal structure of the B30.2/SPRY domain has been solved and identified a conserved peptide-binding site in the vicinity of FMF-associated mutations (115–117). Putative binding partners have not been found, however it is unlikely that the binding pocket directly recognizes PAMPs. Solving the crystal structure of pyrin, ideally at different states of activation and in association with binding partners, such as 14-3-3 protein, PKN1/2 or ASC, could help to answer this question. Recent advances in cryo-electron microscopy may facilitate these studies (118, 119).

As discussed earlier, the binding of PKN1 to murine pyrin fused to the wildtype human B30.2 domain is strongly reduced relative to PKN1 binding to wildtype murine pyrin lacking the B30.2 domain. This finding indicates that the B30.2 domain regulates pyrin phosphorylation/inhibition and suggests that murine pyrin, which lacks B30.2 domain, mostly exists in an inhibited state in the cell. This raises the question, how the pyrin inflammasome can be activated in mice. Park et al. (70) propose the presence of phosphatases that dephosphorylate pyrin to release it from its inactive state. It is also possible that mouse pyrin interacts with a yet to be identified protein that fulfills similar functions as the B30.2 in human and therefore compensates for the absence of this protein domain.

Still little is known about the triggers that cause the outbreak of autoinflammatory attacks. Different factors have been associated with disease flares including infection, trauma, physical and emotional stress, menstruation, and exposure to cold (120–122). The observation that elevated levels of cAMP trigger pyrin inflammasome activation through repression of RhoA might provide a first hint toward understanding the underlying mechanism (70, 123). The cAMP/PKA signaling pathway is an important cellular integrator for a variety of different signals, including hormones and neurotransmitters. These signals bind and stimulate G protein-coupled receptors that subsequently trigger cAMP production (124). In 2015, a study demonstrated the direct association between cAMP/PKA signaling pathway activity and stress-induced behavioral responses (125). Loss of cyclin-dependent protein kinase 5 in the forebrain of mice induced elevated cAMP concentrations and PKA activation in striatal neurons and also affected the behavioral responses to acute or chronic stress. Conversely, it is possible that physical and/or emotional stress affects cAMP/PKA signaling and consequently induces inflammation. The question of what explains the fluctuation in inflammation and what is natural progression toward termination of these episodes of inflammation in FMF and other autoinflammatory disorders remains quite relevant.

A better understanding of the molecular mechanisms that regulate pyrin and other intracellular inflammasomes will ultimately guide development of new therapies for patients with immune dysregulation and other diseases that may benefit from modulations in inflammatory and immune responses.

## Author Contributions

All authors listed have made a substantial, direct and intellectual contribution to the work, and approved it for publication.

### Conflict of Interest Statement

The authors declare that the research was conducted in the absence of any commercial or financial relationships that could be construed as a potential conflict of interest.
